# Room-temperature-superconducting *T*_*c*_ driven by electron correlation

**DOI:** 10.1038/s41598-021-88937-7

**Published:** 2021-05-14

**Authors:** Hyun-Tak Kim

**Affiliations:** Metal-Insulator-Transition & Quantum Lab., Electronics and Telecommunications Research Institute, Daejeon, 34129 South Korea

**Keywords:** Physics, Condensed-matter physics

## Abstract

Room-temperature-superconducting *T*_*c*_ measured by high pressure in hydrides can be theoretically explained by a Brinkman–Rice (BR)–Bardeen–Cooper–Schrieffer (BCS) *T*_*c*_ combining both the generalized BCS *T*_*c*_ and the diverging effective mass, m*/*m* = 1/(1 − (*U*/*U*_*c*_)^2^), with the on-site Coulomb interaction *U* in the BR picture. A transition from *U* in a correlated metal of the normal state to *U*_*c*_ in the superconducting state can lead to superconductivity, which can be caused by volume contraction induced by high pressure or low temperature.

## Introduction

Since 1911, Onnes’s discovery of the superconductivity phenomenon of zero resistance in Hg, the continues efforts have been made to create and find a room temperature superconductor possessing an intriguing scientific and technological potential. Ashcroft predicted that the room-temperature *T*_*c*_ can be achieved for hydrogen solid metal with an extremely high Debye temperature given as inversely proportional to root hydrogen mass ${\upomega }_{Debye} \propto 1/\sqrt {M_{Hydrogen-mass} }$^1^. In 1935, Wigner and Huntington claimed that at a pressure of 25 gigapascals (GPa), solid molecular hydrogen would turn into a metal^2^. Silvera and Dias managed to turn hydrogen to metallic at a pressure of 495 GPa, well beyond the 360 GPa of Earth’s core^3^. In 1970, Satterthwaite & Toepke first observed superconductivity of *T*_*c*_ ≈ 8.05 ~ 8.35 K in the hydrides and deuterides of thorium with H-or D-to-metal atom ratios of 3.60–3.65^4^. They asserted that these materials are apparently type-II superconductors with *H*_*c2*_ of the order of 25–30 kg at 1.1 K^4^. In 2008, a hydride, SiH_4_, revealed the metallic characteristic at 50 GPa and superconductivity of *T*_*c*_ ≈ 17 K at 100 GPa^5^.

From 2005, the high *T*_*c*_ was observed at 203 K and 150 GPa for H_3_S^6^, at 250 ~ 260 K and 180–200 GPa for LaH_10_^7^, at 287 K and 274 GPa for a H–S–C compound^8^, and over onset 500 K for a LaH_10_ superhydride^9^. The first-principle calculations revealed a large density of states at the Fermi energy^10,11^. The isotope shifts of α = 0.50 ~ 0.35 (*T*_*c*_ *≈* *M*^−*α*^) measured for D_2_S^6^, α = 0.465 calculated by the first-principle approximation for LaD_10_^12^, and α = 0.4 experimentally evaluated for YD_6_^13^, suggested that the electron–phonon interaction such as the BCS (Bardeen–Cooper–Schrieffer) *s*-wave superconductor^6,12^ is the pairing mechanism of superconductivity.

**Figure 1:**
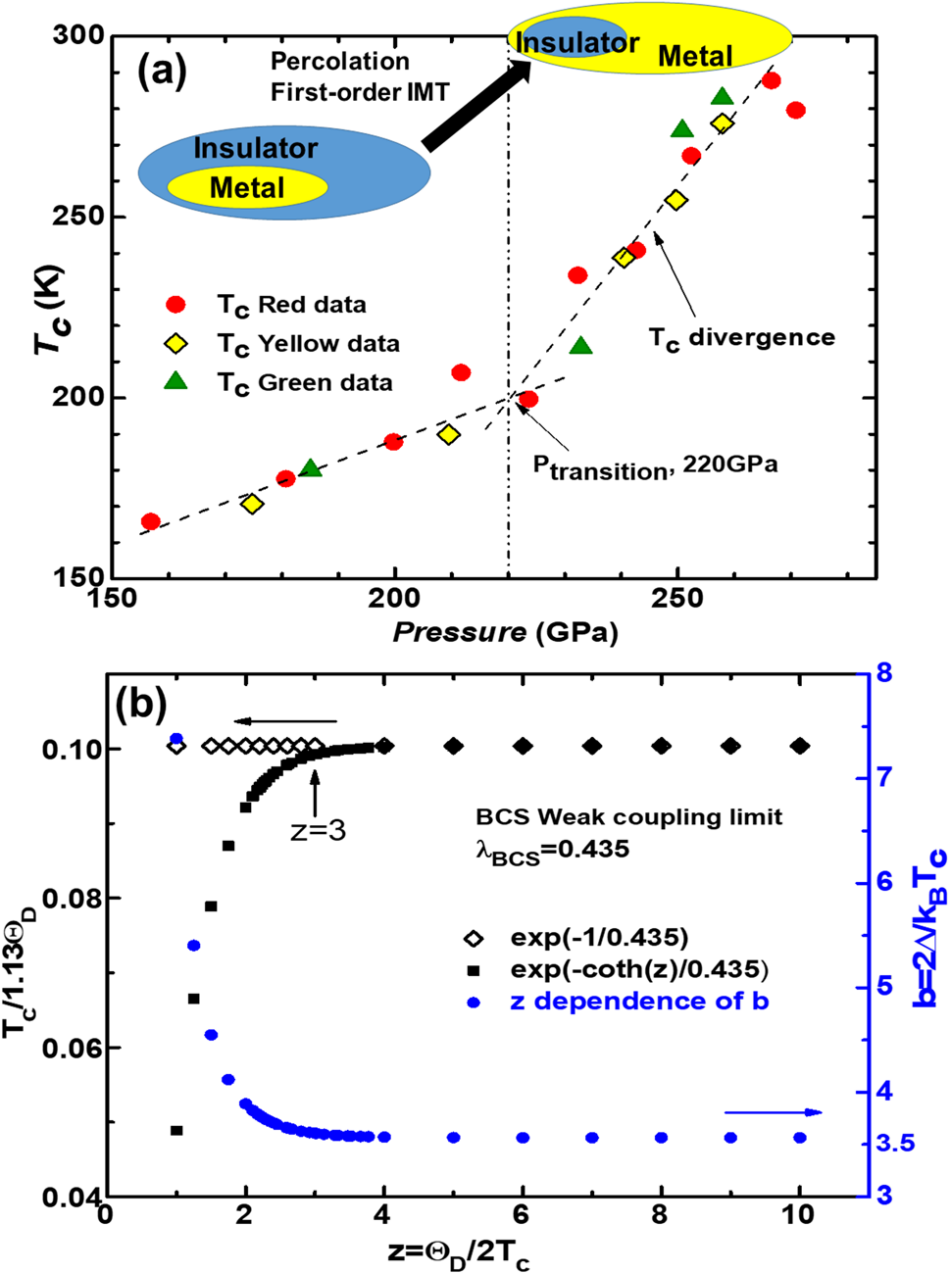
(**a**) Experimental data for the room-temperature *T*_*c*_ as a function of applied pressure^8^, which shows the *T*_*c*_ divergence over *P*_*transition*_ = 220 GPa. The data were extracted in the paper^8^. The insulator–metal transition undergoes the first-order percolation (i.e., an increase in band filling) with increasing pressure (Inset). (**b**) A comparison of the weak coupling *T*_*c*_ (empty diamond) and the generalized *T*_*c*_ (filled square) in BCS theory. The coupling constant, *b* = 2*Δ*/*k*_*B*_*T*_*c*_, (blue ball) between the generalized energy gap and the generalized *T*_*c*_ has no restriction on the magnitude of λ_BCS_. The *T*_*c*_ rapidly decreases below z = 3, which indicates that the generalized *T*_*c*_ does not explain the high *T*_*c*_.

A particular feature of hydrides is a *T*_*c*_ divergence observed above a transition pressure, *P*_*transition*_, which leads to room-temperature superconductivity^8,14,15^, as shown in Fig. 1a. The *T*_*c*_ rise with the applied pressure is gradual below *P*_*transition*_ and sharp over *P*_*transition*_. The gradual *T*_*c*_ rise is attributed to the small increase of the metal phase in the coexistence state of metal and insulator phases, while the sharp *T*_*c*_ rise results from the nearly single metal phase formed by the first-order insulator–metal transition (IMT)^16,17^; this is due to the percolation phenomenon. The IMT is not accompanied by any structural phase transition^6,18^. The IMT-percolation layout is shown in Fig. 1, which indicates that hydrides are the first-order IMT material undergoing percolation with increasing doping (or band filling), such as VO_2_ with inhomogeneity in the IMT process. This process implies hydrides are correlated materials. The first-order phenomenon has also been previously reported^19^.

Regarding the room-temperature *T*_*c*_, it may not be explained by the weak coupling BCS *T*_*c*_ with the electron–phonon coupling constant, λ ≤ 0.435, which describes the low-*T*_*c*_ superconductivity^20^. As an alternative, the strong-coupling McMillan *T*_*c*_^21^ and the Allen-Dynes *T*_*c*_^22^ without a restriction of the magnitude of λ have been suggested, although a max *λ*_*Migdal*_ *≡* *N*(0)*V*_*Migdal*_ ≤ 1.5 has been given^19^. They are based on the Eliashberg formalism utilizing the increase in the Cooper-pair potential *V*_*Migdal*_ with strong coupling^23^ and not the density of states *N*(0), the screened Coulomb repulsive potential *μ*, and the double potential well structure. *μ* depends on the number of carriers and is smaller in magnitude than the on-site short range repulsive Coulomb interaction, *U*. However, in the case of hydrides with a high Debye energy (*ћω*), due to the increase in the retarded Coulomb pseudo-potential, μ^*^ = *μ*/(1 + *μln*(*E*_*F*_/*ћω*)) derived in conditions of *λ* << 1 and *μ* << 1^24^, caused by a large deviation of *ln*(*E*_*F*_/*ћω*) > 1 in μ^*^, the exponential parts in the McMillan *T*_*c*_ and the Allen-Dynes *T*_*c*_ become much smaller than that obtained in BCS theory (see “Methods”). Although Allen-Dynes *T*_*c*_, with *ћω*_*log*_/1.2, an average of the phonon energy, different from *ћω*/1.45 as the prefactor of the McMillan *T*_*c*_, is accurate at a small μ^*^ value^25^, the *T*_*c*_ declines. This is due to the decrease in the exponential part in the *T*_*c*_ formula which is attributed to an increased value of μ^*^ caused by a large Debye energy (see “Methods”)^26^. A comparison of the BCS *T*_*c*_ and the *T*_*c*_s based on the Eliashberg formalism is shown^25^. Furthermore, an *T*_*c*_* ∝ exp* [− 1/(*λ*-μ^*^)] derived in *λ* << 1 and μ^*^ << 1 on the basis of Elisahberg formalism^24^ does not rise to room temperature, because *λ′* = *λ* − μ^*^ decreases with increasing μ^*^ for hydrides. Therefore, the *T*_*c*_s do not reach room temperature.

Subsequently, Migdal’s theory^23^ revealed that the increase in *λ*_*Migdal*_, as strong coupling, results in the decrease in sound velocity proportional to the Debye energy, leading to the decrease in *T*_*c*_. This finding indicates that a strong coupled model cannot explain the high *T*_*c*_. Moreover, an exceedingly large *λ* = 6.2 was evaluated from experimental values using the McMillan *T*_*c*_ for YH_6_^27^, which is much larger than the calculated value (λ = 1.71 ~ 2.24)^13^. The Eliashberg formalism does not fit the isotope effect^11^. Bogoliubov calculated the electron–phonon interaction by introducing the screened Coulomb repulsive interaction between electrons^28^, concluding that the screened Coulomb interaction plays little role in inducing superconductivity because the magnitude of the electron–phonon interaction is largely reduced by the Coulomb interaction. Thus, no theory is available to explain the high *T*_*c*_. To enhance the *T*_*c*_, the magnitude of density of states *N*(0) rather than the electron–phonon interaction should be increased. A BCS-based *T*_*c*_ that uses large *N*(0) as a function of band filling is needed.

In this report, we confirm the rise in *T*_*c*_ to room temperature by demonstrating the *T*_*c*_ divergence over *T*_*ransition*_ using a proposed BCS theory supported by the Brinkman-Rice picture^29^, with the diverging effective mass contributing to the density of states for a strongly correlated metal with *U*/*U*_*c*_ = *κ*_*BR*_ ≈ 1 (≠ 1). We reveal a fundamental cause of the electron–phonon interaction for superconductivity. The cause has remained obscure since the discovery of Onnes’s superconductivity in 1911, despite the development of BCS theory.

## Derivations of superconducting-***T***_***c***_ formulas

### Generalized energy gap and ***T***_***c***_ in BCS theory

To overcome the weak coupling limitation of λ ≤ 0.435 in BCS theory, the energy gap of the Cooper pair and *T*_*c*_ need to be generalized. We find a generalized energy gap of the Cooper pair, a generalized *T*_*c*_, and a generalized coupling constant between the energy gap and *T*_*c*_ without any restrictions in BCS theory. The energy gap, *ε*_*g*_ = *Δ*, of Eq. (2.40) in BCS theory^20^ is derived using *sinh*(*x*) = (*e*^*x*^ − *e*^−*x*^)/2 as follows:

1 $$\Delta = \frac{\hbar \omega }{{{\text{sinh}}\left[ {\frac{1}{{\lambda_{BCS} }}} \right]}} = \frac{{2\hbar \omega {\text{exp}}\left[ { - \frac{1}{{\lambda_{BCS} }}} \right]}}{{1 - {\text{exp}}\left[ { - \frac{2}{{\lambda_{BCS} }}} \right]}},$$

where *ћω* is the Debye’s phonon vibration energy, *λ*_*BCS*_ = *N*(0)*V*_*e*-*ph*_ is the electron–phonon coupling constant when the electron correlation is not considered, *N*(0) is the density of Bloch states of one spin per unit energy at the Fermi surface *E*_*F*_, and *V*_*e*-*ph*_ is a constant matrix element of the electron–phonon pair energy. Equation (1), satisfied with *λ*_*BCS*_ ≠ ∞, has a divergence in the denominator and has no restrictions on the magnitude of *λ*_*BCS*_. In the case of *λ*_*BCS*_ ≤ 0.435, (which is the weak coupling limit confirmed by this author), Eq. (1) is reduced to the famous BCS energy gap, 2*ћω*exp(− 1/*λ*_*BCS*_), by disregarding the extremely small value of exp(− 2/*λ*_*BCS*_). At *λ*_*BCS*_ = 0.435, Δ/2*ћω* ≈ 0.1 is in the weak coupling limit of BCS theory. At *λ*_*BCS*_ > 0.435, the divergence of [1 − exp(− 2/*λ*_*BCS*_)]^−1^ contributes to the enhancement of the energy gap. The derivation of Eq. (1) is given in the Supplementary Information.

As for superconducting *T*_*c*_, the *T*_*c*_ equation of Eq. (3.28) in BCS theory^20^ is generalized without an approximation of a condition, *T*_*c*_ << *ћω*/*k*_*B*_ = *Θ*_*D*_, and any restriction on *λ*_*BCS*_, calculated as

2 $$T_{c} = C\left( z \right)\Theta_{D} exp\left[ { - \frac{\coth \left( z \right)}{{\lambda_{BCS} }}} \right],$$

3 $$\approx { }1.13\Theta_{D} exp\left[ { - \frac{\coth \left( z \right)}{{\lambda_{BCS} }}} \right],$$

where *z* = *Θ*_*D*_/*2T*_*c*_ is given, and $C\left( z \right) \equiv \frac{1}{2}exp\left[ { - coth\left( z \right)\int_{0}^{z} {(ln\left( z \right)/cosh^{2} z)} dz} \right]$ is defined^30^. Here, to be the maximum *T*_*c*_ in Eq. (2), z should be ∞ in the function of *C*(*z*), after which *coth*(*z*) = 1 and max $C\left( z \right) \equiv \frac{1}{2}exp\left[ { - \int_{0}^{\infty } {({\text{ln}}\left( z \right)/cosh^{2} z)} dz} \right] = \left( {\frac{{2e^{\gamma } }}{\pi }} \right)\approx 1.13$ are obtained, where γ ≈ 0.577 is the Euler constant. The derivation of Eq. (2) is given in the Supplementary Information. The *T*_*c*_ decreases with a decreasing z below z = 3, as shown in Fig. 1b. This phenomenon deviates from the limitation of the weak coupling BCS theory in which *T*_*c*_ is defined as over z = 3.

Moreover, the relation between the generalized energy gap *Δ* in Eq. (1) and the generalized *T*_*c*_ in Eq. (3) is given as

4 $$b = \frac{2\Delta \left( 0 \right)}{{k_{B} T_{c} }} = 3.54\frac{{exp\left( {\frac{1}{{\lambda_{BCS} }}\left[ {\coth \left( z \right) - 1} \right]} \right)}}{{1 - exp\left( { - \frac{2}{{\lambda_{BCS} }}} \right)}}.$$

The coupling constant, *b*, rapidly increases below z = 3 irrespective of a value of *λ*_*BCS*_, as shown in Fig. 1(b), and it also increases over *λ*_*BCS*_ ≈ 0.435.

### Superconducting *T*_*c*_ driven by electron correlation

High-*T*_*c*_ superconductors with z < 3 have the *T*_*c*_ enhancement. In contrast, the *T*_*c*_ in Eq. (3) decreases, as shown in Fig. 1b. This means that Eq. (3) does not account for the increased *T*_*c*_. Thus, to raise *T*_*c*_, as a new concept, we assume the existence of the on-site Coulomb repulsive interaction (or correlation), *U*, between free electrons at the Fermi surface in a strongly correlated metal with *U*/*U*_*c*_ = *κ*_*BR*_ ≈ 1 (≠ 1) where *U*_*c*_ is a critical Coulomb interaction. The assumption is based on the first-principle calculations^10,11^, the divergence of the effective mass near the optimal doping^31–33^, and a suggestion that the strong correlation needs to be introduced^34^. The mass of carriers (quasiparticles) in the correlated metal is much heavier than that in the metal of BCS theory. As a result, the kinetic energy, *ε*_*k*_, of the carriers, as expressed as *ε*_*k*_ = *ε*_*BCS*_(1 − (*U*/*U*_*c*_)^2^)^2^ with the effective mass of carriers m^*^ = *m*/(1 − (*U*/*U*_*c*_)^2^), is reduced with increasing *U*^29^. The kinetic energy does not contribute to the electron–phonon interaction^35^. Although *ε*_*BCS*_ is replaced by *ε*_*k*_, the Hamiltonian and the *T*_*c*_-formula form in BCS theory are not changed^35^. The BCS *T*_*c*_ equation was also solved by the Green function method^36^. The effect of the heavy mass of the carriers is independently compensated in the density of states for the *T*_*c*_ formula. Additionally, the inhomogeneity effect intrinsically appearing in the strongly correlated materials needs to be considered, which has been previously developed^32,33^.

**Figure 2:**
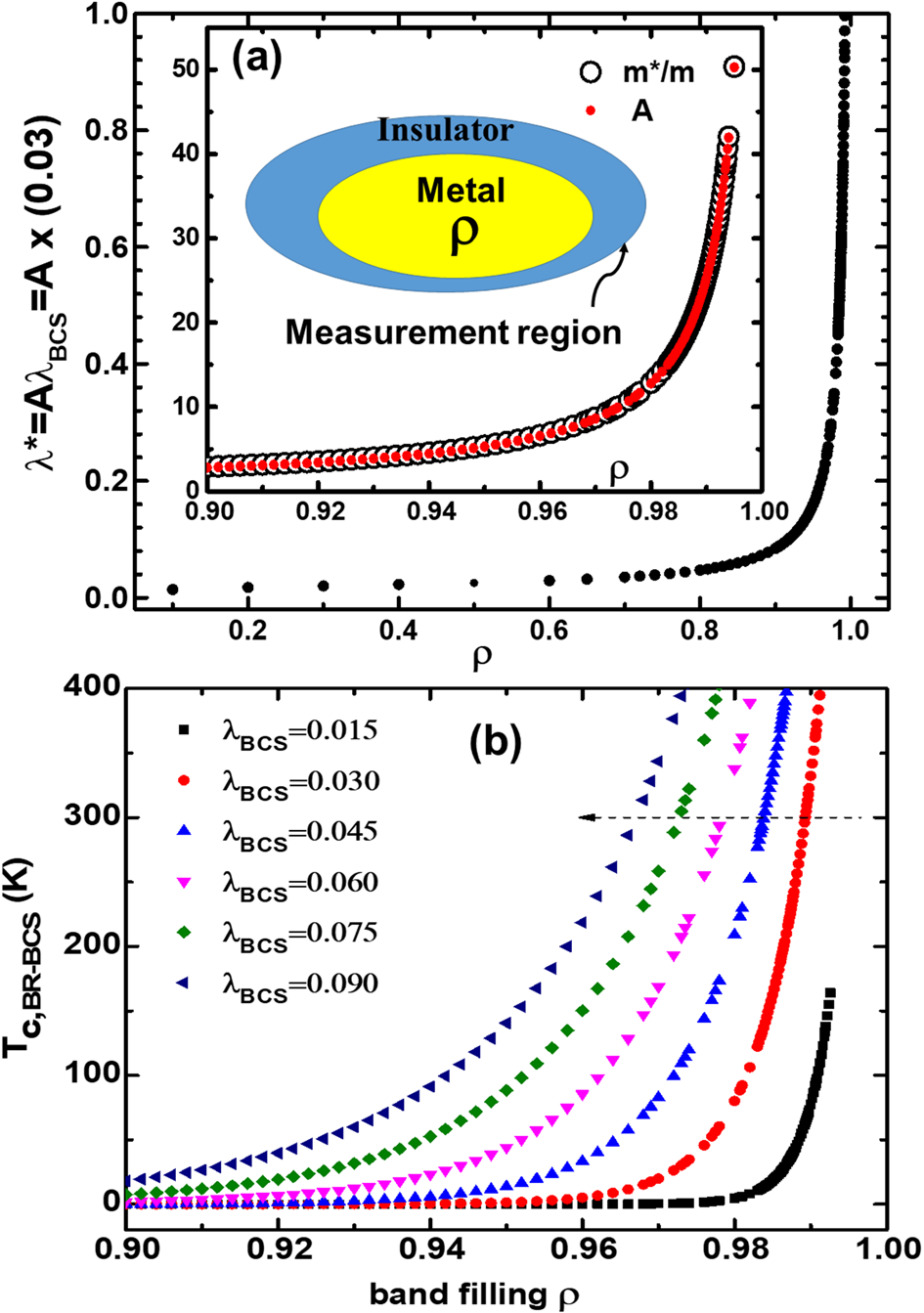
(**a**) A divergence of an effective electron–phonon-coupling constant, λ^*^ = *Aλ*_*BCS*_ with *λ*_*BCS*_ = 0.03, is shown as a function of band-filling *ρ*, where *A* = *N*(0)*/*N*(0) = *ρ*^(1/3)^/(1 − *κ*_*BR*_^2^*ρ*^4^) at *κ*_*BR*_ *≈* 1 is a ratio of the 3D density of states, and *N*(0) is the 3D density of states. The inset displays the divergences of the effective mass, m*/*m* = 1/(1 − *ρ*^4^)^32,33^, and the ratio *A*. In the inset, the layout of the inhomogeneous mixed phase with a correlated metal (*κ*_*BR*_ *≡* *U*/*U*_*c*_ ≈ 1 (≠ 1)) and insulator phases in the measurement region is also depicted. (**b**) The *λ*_*BCS*_ dependence of the BR-BCS *T*_*c*_ is shown. Here, the *Θ*_*D*_ = 1250 K in Eq. (6) was used. As *λ*_*BCS*_ increases, *T*_*c*_ increases at a constant *ρ*. At a constant *T*_*c*_, as *λ*_*BCS*_ increases, *ρ* decreases but λ^*^ does not change.

Then, Eq. (3) is newly defined as follows;

5 $$T_{c,BR - BCS} \approx 1.13\Theta_{D}^{*} exp\left[ { - \frac{\coth \left( z \right)}{{\lambda_{BCS}^{*} }}} \right],$$

6 $$= 1.13\rho^{\frac{1}{3}} \Theta_{D} exp\left[ { - \frac{{{\text{coth}}\left( z \right)}}{{\left( {\frac{{\rho^{\frac{1}{3}} }}{{1 - \kappa_{BR}^{2} \rho^{4} }}} \right)\lambda_{BCS} }}} \right],$$

when *ρ* ≈ 1 from Eq. (6),

7 $$= 1.13\Theta_{D} exp\left[ { - \frac{{{\text{coth}}\left( z \right)}}{{\left( {\frac{1}{{1 - \kappa_{BR}^{2} }}} \right)\lambda_{BCS} }}} \right],$$

when *coth*(*z*) = 1 over z = 3 from Eq. (6),

8 $$= 1.13\rho^{\frac{1}{3}} \Theta_{D} exp\left[ { - \frac{1}{{\left( {\frac{{\rho^{\frac{1}{3}} }}{{1 - \kappa_{BR}^{2} \rho^{4} }}} \right)\lambda_{BCS} }}} \right],$$

where *Θ*_D_^*^ = *ρ*^1/3^*Θ*_*D*_ is an effective Debye temperature, λ^*^ *≡* *Aλ*_*BCS*_ is an effective coupling constant, and *A* *≡* *N*(0)^*^/*N*(0) = *ρ*^1/3^/(1 − *κ*_*BR*_^2^*ρ*^4^) is a ratio of an effective 3D-density of states, *N*(0)^*^ ∝ m^*^n^1/3^, at *E*_*F*_. In the two dimensional case, *N*(0)^*^ ∝ m^*^ is given. The *λ*_*BCS*_ is a constant, which is indefinite and must be extremely small. An effective mass of quasiparticles is given as m^*^/*m ≡ *1/(1 − (*U*/*U*_*c*_)^2^) = 1/(1 − *ρ*^4^) from *U*/*U*_*c*_ = *κ*_*BR*_*ρ*^2^ and, the correlation strength, 0 < *κ*_*BR*_ < 1 and, here, *κ*_*BR*_≈1 (or 0.999…, not one)^29,32,33^ (Fig. 2a). A carrier density at *E*_*F*_, *n* = *ρn*_*tot*_, is the extent of the metal region, 0 < *ρ* = *n*/*n*_*tot*_ < 1 is the band-filling factor (or the normalized carrier density), and *n*_*tot*_ is the number of all atoms in the measurement region^32,33^. *ρ* can be obtained from the Hall-effect experiment or the integral of the optical conductivity. *ρ*^1/3^ in *Θ*_D_^*^ comes from the number of phonons in the phonon energy of lattices in the superconducting region (or metal phase over *T*_*c*_) (inset in Fig. 2a). m^*^ = *m*/(1 − *ρ*^4^) is obtained by applying an effective Coulomb energy, *U*/*U*_*c*_ = *κ*_*BR*_*ρ*^2^ and *κ*_*BR*_ *≈* 1, deduced in an inhomogeneous system to the Brinkman-Rice(BR) picture explaining the correlation effect in correlated metals formed by the impurity-driven IMT^37–39^, which is an average effect (or measurement effect) of the true effective mass, m^*^ = *m*/(1 − *κ*_*BR*_^2^) at *ρ* = 1^32,33^. The *λ*_*BCS*_ dependence of *T*_*c,BR*-*BCS*_ is shown in Fig. 2b. A large *T*_*c*_ change occurs in a small *ρ* variation near the half-filling *ρ* ≈ 1, confirming the presence of a divergence in the *T*_*c*_ formula. Moreover, when the *λ*_*BCS*_ value is slightly changed, *ρ* also varies. At a constant *T*_*c*_, as *λ*_*BCS*_ increases, *ρ* decreases, but λ^*^ does not change. Moreover, the physical meaning of the *T*_*c,BR*-*BCS*_ of Eq. (6) indicates an experimentally measured local *T*_*c*_ in the measurement region, which is an average (measurement effect) of the large intrinsic true *T*_*c*_ of Eq. (7) expressed by the true effective mass, m^*^ = *m*/(1 − *κ*_*BR*_^2^), at *ρ* ≈ 1 in the BR picture^29^ (see Supplementary Information). The intrinsic true *T*_*c*_ of Eq. (7) is given as a function of *κ*_*BR*_ by applying *ρ* ≈ 1 into Eq. (6), which has a large diverging value near *κ*_*BR*_ = 1. The true *T*_*c*_ is constant determined at a given *κ*_*BR*_ *≈* 1 (*≠ *1). The observed energy gap is obtained by replacing *ћω* and *λ*_*BCS*_ in Eq. (1) with *k*_*B*_*Θ*_D_^*^ and λ^*^, respectively. The coupling constant, *b*, is determined by substituting *λ*_*BCS*_ in Eq. (4) with λ^*^. Moreover, in the case of over z = 3, *coth*(*z*) in Eqs. (2) ~ (7) can be replaced with one and Eq. (8) becomes a BR-BCS *T*_*c*_.

Furthermore, we briefly note the physical meaning of *ρ*. For instance, it means that, in the case of *ρ* = 1, the whole measurement region is filled with a correlated metal of one electron per atom in real space, (inset in Fig. 2a), and the band is half-filled in k-space. In the case of *ρ* = 0.5, 50% of the measurement region is the metal in real space. Moreover, a condition of *ρ* = 1 is not defined due to the inability of *U*/*U*_*c*_ = 1 at m*/*m* = 1/(1 − (*U*/*U*_*c*_)^2^) in the BR picture^29^. That is, neither the point of *ρ* = 1 nor half filling is attainable. This indicates that the correlated material is intrinsically inhomogeneous, which is the characteristic of the correlated material.

## Results and discussions

In the superconducting state, the electron-phonon interaction, *V*_*e*-*ph*_, forming the Cooper pair (pairing in k-space, time-reversed states) in BCS theory is fixed as a constant in real space and k space. This indicates the Cooper pair is a pair in real space (so called bipolaron), such as the pair potential *Δ*(*r*) proportional to *V*_*e*-*ph*_ = − *V*(*r*_1_*,r*_*2*_)*δ*(*r*_1_–*r*_*2*_) suggested in the Bogolubov–de Genes (BdG) theory^28,40,41^. The BdG theory derives the BCS formula for superconductors not only without impurities explained by BCS theory but also with nonmagnetic impurities both making a boundary between metal and nonmetal and not suppressing the superconducting gap^42^; this is an extension of the BCS theory. For a logical deduction of the constant, we consider an intersite charge-density-wave (CDW) potential as an electron-phonon interaction, *V*_*CDW*_ = − (*g*^2^/*2Mω*^2^)*δq*^2^, such as the CDW with a charge disproportionation between nearest neighbor sites, *δq* ≡ *δ*(*q*_*i*_–*q*_*j*_) = 2*e*, of BaBiO_3_ with the set Bi^3+^(6s^2^, the two electrons form bipolaron as a real-space pair) and Bi^5+^(6s^0^)^43,44^ (necessarily see “Methods”); the *V*_*CDW*_ has an immobile bipolaron in real-space, thus indicating a set of both a paired occupied state (bipolaron) with two electrons on a site and an unoccupied state without electron at the nearest neighbor site. A range of the intersite CDW potential that reaches out in real space is within two lattice constants of 6~10 Å when the lattice constant in a metal is considered 4 ± 1 Å. Experimental evidence of the CDW in oxide superconductors is a distortion of octahedral structure observed just below *T*_*c*_^45,46^ and discontinuity^27^ of the bulk modulus at *T*_*c*_. For superconductivity, when the CDW potential is introduced, the on-site critical Coulomb energy *U*_*c*_ in the BR picture should be present at the bipolaron, then, as a nonlocal potential, *V*_*e*-*ph*_ = *V*_*CDW*_ + *U*_*c*_ < 0 is considered a constant, because *V*_*CDW*_ and *U*_*c*_ are determined as fixed values in a crystal. Since *U*_*c*_ is very large and constant, *V*_*e*-*ph*_ becomes extremely small or can approach but not reach zero; this explains why *λ*_*BCS*_ = *N*(0)*V*_*e*-*ph*_ should be small; further, *N*(0) is also small in an uncorrelated metal^47^ (see “Methods”). Then, the bipolaron can tunnel through the CDW potential to the next site; the supercurrent flows, which indicates the bipolaron has changed into the mobile Cooper pair in k-space (so called the mobile bipolaron) due to the *U*_*c*_. Moreover, in the case of a strong coupling with a large *V*_*e*-*ph*_, the Cooper pair can be trapped. Thus, we assert that *U*_*c*_ leads to superconductivity and that, although λ^*^ in Eq. (6) is large (over one) (see Ti-2223 and Hg-1223 in Table 1), *T*_*c*_ of Eq. (6) is into weak coupling due to small *V*_*e*-*ph*_ in *λ*_*BCS*_ (Table 1).

**Table 1:** When experimental data in Fig. 1 are confirmed by Eq. (6), the obtained parameters are evaluated by the following formulas; m^*^/*m* *≡* 1/(1 − (*U*/*U*_*c*_)^2^) = 1/(1 − *κ*_*BR*_^2^*ρ*^4^) *≈* 1/(1 − *ρ*^4^) at *κ*_*BR*_ *≈* 1 (≠ 1), *A* = *N*(0)*/*N*(0) = *ρ*^1/3^/(1 − *ρ*^4^)*,* and λ^*^ = *Aλ*_*BCS*_*,* *Θ*_D_^*^ = *ρ*^1/3^*Θ*_*D*_*.*

Materials	Pressure (Gpa)	T_c_ (K)	Θ_D_ (K)	T_c__BRBCS_	z = Θ_D_/2T_c_	coth (z)	ρ	m*/m	A	λ_BCS_	λ*	T_BRBCS_/113Θ*	Δ (meV)	b	References T_c_, Θ_D_
H–S–C (Red)	266.53	287.8	1250	288.7	2.17	1.026	0.9882	21.6	21.5	0.03	0.644	0.20	23.80	3.9	^1,8^
	270.88	279.6	1250	278.8	2.24	1.023	0.9879	21.0	21.0	0.03	0.629	0.20	22.81	3.8	^1,8^
	252.41	267.0	1250	266.3	2.34	1.019	0.9875	20.4	20.3	0.03	0.609	0.19	21.56	3.8	^1,8^
	242.64	240.9	1250	239.5	2.59	1.011	0.9866	19.0	19.0	0.03	0.569	0.17	19.03	3.7	^1,8^
	232.32	233.9	1250	234.0	2.67	1.010	0.9864	18.8	18.7	0.03	0.560	0.16	18.52	3.7	^1,8^
	*2*11.68	207.0	1250	207.3	3.02	1.005	0.9854	17.5	17.4	0.03	0.523	0.15	16.16	3.7	^1,8^
	223.63	199.7	1250	199.7	3.13	1.004	0.9851	17.2	17.1	0.03	0.512	0.14	15.52	3.6	^1,8^
	199.73	187.8	1250	187.7	3.33	1.003	0.9846	16.6	16.5	0.03	0.496	0.13	14.52	3.6	^1,8^
	180.72	177.6	1250	178.5	3.52	1.002	0.9842	16.2	16.1	0.03	0.484	0.13	13.76	3.6	^1,8^
	156.82	165.8	1250	163.4	3.77	1.001	0.9835	15.5	15.4	0.03	0.463	0.12	12.54	3.6	^1,8^
(Yellow)	257.84	275.9	1250	275.7	2.27	1.022	0.9878	20.9	20.8	0.03	0.624	0.19	22.49	3.8	^1,8^
	249.7	254.7	1250	254.2	2.45	1.015	0.9871	19.8	19.7	0.03	0.590	0.18	20.39	3.8	^1,8^
	240.46	238.8	1250	236.6	2.62	1.011	0.9865	18.9	18.8	0.03	0.564	0.17	18.77	3.7	^1,8^
	209.5	189.9	1250	190.1	3.29	1.003	0.9847	16.7	16.6	0.03	0.499	0.13	14.71	3.6	^1,8^
	174.74	170.7	1250	169.7	3.66	1.001	0.9838	15.8	15.7	0.03	0.472	0.12	13.05	3.6	^1,8^
(Green)	257.84	282.9	1250	281.9	2.21	1.024	0.9880	21.2	21.1	0.03	0.634	0.20	23.13	3.8	^1,8^
	250*.78*	273.9	1250	272.4	2.28	1.021	0.9877	20.7	20.6	0.03	0.619	0.19	22.17	3.8	^1,8^
	232.86	213.9	1250	212.3	2.92	1.006	0.9856	17.7	17.7	0.03	0.530	0.15	16.61	3.7	^1,8^
	185.06	180.1	1250	178.5	3.47	1.002	0.9842	16.2	16.1	0.03	0.484	0.13	13.76	3.6	^1,8^
H_3_S		200.8	1560	201.4	3.88	1.001	0.9834	15.4	15.4	0.03	0.461	0.11	15.45	3.6	^50^
D_3_S		155.0	869	155.0	2.80	1.007	0.9860	18.2	18.2	0.03	0.545	0.16	12.19	3.7	^50^
LaH_10_		240.0	1310	239.4	2.73	1.009	0.9862	18.5	18.4	0.03	0.552	0.16	18.88	3.7	^50^
LaH_x_		207.0	1675	208.1	4.05	1.001	0.9831	15.2	15.1	0.03	0.453	0.11	15.95	3.6	^50^
LSCO		50.0	383	49.4	3.83	1.001	0.9834	15.4	15.4	0.03	0.461	0.11	3.79	3.6	^53^
YBCO		91.0	426	90.7	2.34	1.019	0.9875	20.4	20.3	0.03	0.609	0.19	7.35	3.8	^53^
Bi-2223		110.0	334	110.4	1.52	1.101	0.9915	29.8	29.7	0.03	0.891	0.29	10.45	4.4	^53^
Ti-2223		120.0	226	120.7	0.94	1.359	0.9958	59.9	59.8	0.03	1.794	0.47	16.58	6.4	^53^
Hg-1223		133.0	200	132.3	0.75	1.572	0.9974	96.5	96.4	0.03	2.893	0.58	24.42	8.6	^53^
Nb		9.2	184	9.1	9.98	1.000	0.9759	10.8	10.7	0.03	0.320	0.04	0.69	3.5	^54^
Pb		7.3	86	7.2	5.92	1.000	0.9800	12.9	12.8	0.03	0.384	0.07	0.55	3.6	^54^
Ta		4.4	246	4.5	28.08	1.000	0.9680	8.2	8.1	0.03	0.243	0.02	0.34	3.5	^54^
Hg		4.1	69	4.1	8.37	1.000	0.9773	11.4	11.3	0.03	0.339	0.05	0.31	3.5	^54^
Sn		3.7	180	3.7	24.39	1.000	0.9688	8.4	8.3	0.03	0.249	0.02	0.28	3.5	^54^
Tl		2.4	100	2.4	21.01	1.000	0.9700	8.7	8.6	0.03	0.259	0.02	0.18	3.5	^54^

*T*_*c,BR*-*BCS*_ given from Eq. (6). The energy gap, *Δ*, is determined by using Eq. (1) substituted by *Θ*_D_* = *ρ*^1/3^*Θ*_*D*_ and λ*. The coupling-constant *b* was obtained by Eq. (4). LSCO is La_1.8x_Sr_0.2_CuO_7-δ_. YBCO is YBa_2_Cu_3_O_7-δ_. Bi-2223 is Bi_2_Sr_2_Ca_2_Cu_3_O_11-δ_. Ti-2223 is Tl_2_Ba_2_Ca_2_Cu_3_O_10+δ_. Hg-1223 is HgBa_2_Ca_2_Cu_3_O_8+δ_. The λ*s are over one for Ti-2223 and Hg-1223, which is attributed to the large effective mass.

Subsequently, the coherence length was known as approximately *ξ*_0_ ≈ 5 Å^34^, within the range of two-lattice constant. The radius of the Cooper pair in real space^48^ was given as *r*_*Cooper*_ _*pair*_ = *πξ*_0_. The coherence length, utilizing both the pair potential *Δ*(*r*) = *Δ*(0) at *r* = 0 calculated from the generalized BdG theory and the effective mass m^*^, was given as $\xi_{0} = \frac{{\hbar {\text{v}}_{{\text{F}}} }}{\pi \Delta \left( 0 \right)} = \left( {\frac{\hbar }{{\pi {\Delta }\left( 0 \right)}}} \right)\sqrt {\frac{{2E_{F} }}{{m^{*} }}} ,$ where *Δ*(0) = 0.2*ħω*_*D*_ and *ξ*_0_ = 0.2*a* for a nano crystal of a size of *a* = 15 nm was evaluated^49^. Moreover, Deloof et al.^49^ stated that the computational effect is reduced by increasing the effective mass and the coupling constant by decreasing the sample size. This author, according to the concept described here, adds that the large effective mass coming from the on-site Coulomb *U* can reduce the coherence length to a short range of two-lattice constant. A model of superconductivity based on the CDW has been reported^44^.

**Figure 3:**
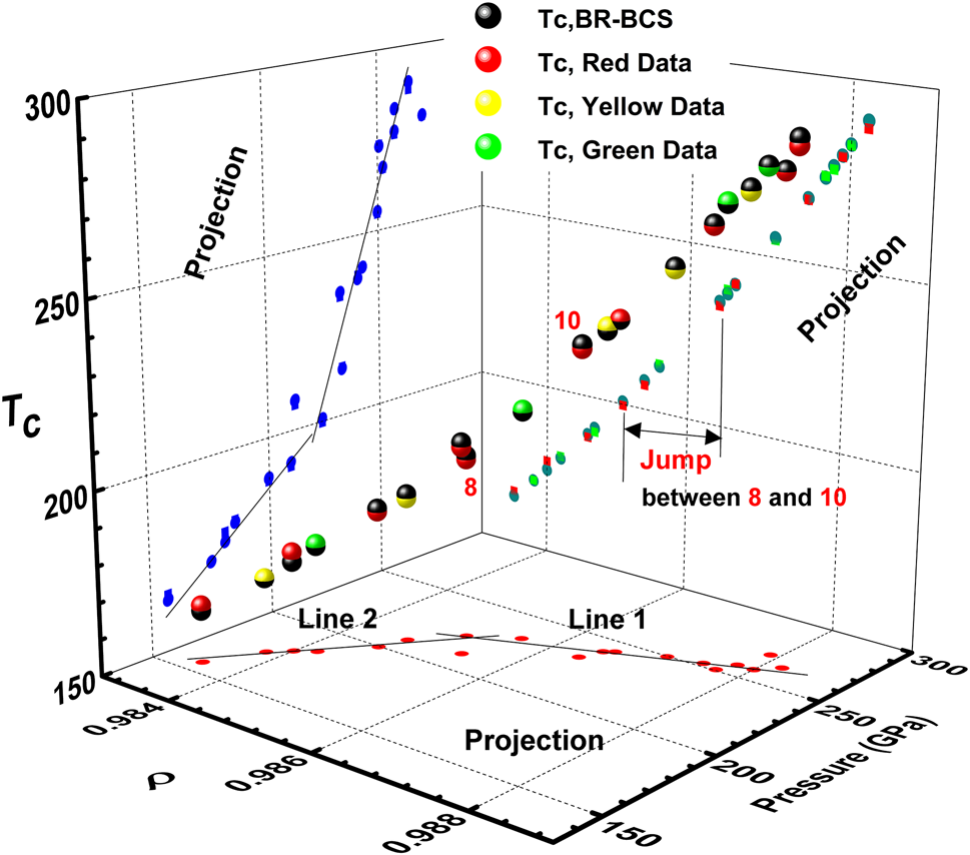
The BR-BCS *T*_*c*_ of Eq. (6) and data in Fig. 1a are drawn together. The *T*_*c*_ calculations cannot be correct, because the Debye temperature, *Θ*_*D*_, is not correct; here *Θ*_*D*_ = 1250 K was predicted in a hydride^1^, which indicates that Eq. (6) approaches the room-temperature *T*_*c*_. The jump in *ρ* is observed as evidence of the first-order IMT. The detailed information is provided in Table 1. At line 1 over *P*_*transition*_ ≈ 220 GPa, the relation between *ρ* and pressure *P* is *P* = 11,759.62*ρ − *11,359.59, where the slope has a standard error of 747.35 and the standard error of the intercept is 737.64. At line 2 below *P*_*transition*_, the relation between *ρ* and pressure is given as *P* = 25,230.15*ρ* − 24,644.25, where the slope has a standard error of 4660.97 and the standard error of the intercept is 4588.50. The slope of line 1 is much larger than that of line 2, revealing the diverging behavior.

We apply the *T*_*c*_ of Eq. (6) to the experimental data for *T*_*c*_ with a transition pressure^8^, using *Θ*_*D*_ *≈* 1250 K in a hydride mentioned by Ashcroft^1^. Note that the *Θ*_*D*_ is not an accurate value because it is not yet known. The *Θ*_*D*_ is used to check whether the *T*_*c*_ of Eq. (6) can rise to room temperature or not. The *T*_*c*_ values in Eq. (6) seem to rise to room temperature, as shown in Fig. 3. A relation of *P* vs. *ρ* is given in the caption of Fig. 3. The obtained parameters are given in Table 1. The obtained λ^*^s are over 0.435, the weak coupling limit of BCS theory. When precisely calculated *Θ*_*D*_*s* for the hydrides of H_3_S, D_3_S, LaH_10_, and LaH_x_ are used^50^, the λ^*^s are also more than 0.435 and less than one (Table 1). We assert that the metallization is accelerated with increasing pressure, which is regarded as the increase in *ρ*. As evidence of the increased metallization induced by the first-order IMT, a jump in *ρ* is observed, as shown in Fig. 3. Furthermore, although λ^*^s are over one for Ti-2223 and Hg-1223 in Table 1, the large λ^*^s are caused by the large effective mass (large density of states) and not a large potential *V*_*e*-*ph*_, such as the strong coupling potential *V*_*Migdal*_ used in the Eliashberg formalism. Moreover, in Table 1, λ^*^ = 0.384 for Pb, known as strong coupling of λ^*^ = 1.12^21^ and 1.55^22^, is less than λ^*^ = 0.435 of the weak coupling limit in BCS theory.

We briefly discuss a process of the IMT and a change in the correlation strength under high pressure. Compound materials are necessarily inhomogeneous and have an impurity level reflecting the semiconducting behavior. When pressure, temperature, strain, and chemical doping, among other energies are applied to the materials, the Mott-indirect IMT occurs by excitation of the impurity bound charges^37–39^. In the underdoped region, as the pressure increases, the extent of the correlated-metal region, *ρ*, increases due to the indirect IMT (percolation). Therefore, in some materials, at low temperatures, superconductivity appears. Decreasing the temperature reduces the size of the unit volume of the correlated metal (i.e., contraction of the unit volume), which causes an increase in the correlation strength. Additionally, applying pressure to the correlated materials leads to metallization as well as contraction of the unit volume, resulting in both an enhanced correlation and an increase of *ρ*. Thus, the density of states as a function of the effective mass diverges near *ρ* = 1 due to strong correlation of a constant value of *κ*_*BR*_ ≡ *U*/*U*_*c*_ ≈ 1 (not one), as shown in Fig. 2a. Thus, the *T*_*c*_ in Eq. (6) rapidly increases, which is the *T*_*c*_ divergence, as shown in Fig. 2b.

Furthermore, in the BCS-based mechanism for all kinds of superconductors, when the correlation effect in the density of states is introduced, the coupling constant, λ_BCS_, should be replaced with λ^*^ = Aλ_BCS_ including the correlation effect. When λ_BCS_ < 0.1 with a small value^47^ (see “Methods”), instead of λ^*^, is applied to Eq. (5), *T*_*c*_ is not obtained; this is a weak point of BCS theory. This finding indicates that superconductivity does not occur without correlation; this is a mathematical discovery. Until now, to explain low-temperature superconductivity, a value near λ_BCS_ = 0.20 ~ 0.30 has been used, which should really be regarded as λ^*^. Moreover, the element superconductors explained by BCS theory should be regarded as correlated metals which are different from pure metals such as Au, Ag, or Cu that do not show superconductivity. The metallization in the element superconductors, including a non-metallic phase of few concentrations considered as impurity, is induced by the impurity-driven indirect IMT. This phenomenon is understood by observing the rise in *T*_*c*_ when pressure is applied to the element superconductors^51,52^, because the pressure effect does not appear in the pure metal crystals. Additionally, Eq. (6) can describe the high *T*_*c*_ of the cuprate superconductors. The λ^*^ values obtained for important cuprate superconductors are given in Table 1. The energy gaps are slightly less than those we observed in the present analysis, which may be attributed to a smaller *Θ*_*D*_. We suspect that the observed *Θ*_*D*_ was averaged to the multi-layered and inhomogeneous cuprate system, not measured on only the CuO_2_-layered plane. Accordingly, we assert that the superconductivity for all kinds of superconductors is caused by a change in the electron correlation that occurs due to the volume contraction induced by strong pressure or low temperature; this indicates that *U* in the correlated metal of the normal state can change to *U*_*c*_ of the condensed superconducting gapped state, which leads to the electron–phonon interaction at *T*_*c*_.

## Conclusion

The *T*_*c,BR*-*BCS*_ with the electron correlation of Eq. (6) accounts for the high *T*_*c*_. It can be applied to all kinds of superconductors, such as element superconductors, compound superconductors, cuprate superconductors, and hydride superconductors, among others. The diverging *T*_*c*_ measured in the hydrides^8^ is responsible for the pressure-driven first-order IMT. Superconductivity can be attributed to the transition of the Bose–Einstein condensation from *U* to *U*_*c*_, which derives from the volume contraction by applied pressure or low temperature.

## Methods

### Evaluation of the strong-coupled-McMillan ***T***_***c***_

μ* *≡* *μ*/(1 + *μln*(*E*_*F*_/*ћω*)) should be satisfied with μ* << 1^21,24^*.* μ* = (1 − 2*α*)^0.5^/*ln*(*Θ*_*D*_/1.45*T*_*c*_) at λ <  < 1 was obtained from neglecting ‘strong-coupling’ correction term^21^. For D_3_S, *α* = 0.50 ~ 0.35 (Isotope effect^6^), *Θ*_*D*_ = 869 K, and *T*_*c*_ = 155 K were determined^50^. For *α* ≈ 0.465^8^, μ* = 0.196 and for *α* ≈ 0.35, μ* = 0.405 are determined. In the case of max λ ≤ 1.5^19^, for the McMillan *T*_*c*_/(0.69*Θ*_*D*_) = *exp*(− [1.04(1 + *λ*)/(*λ *− μ*(1 + 0.62*λ*)]), *T*_*c*_/(0.69*Θ*_*D*_) = 0.0985 ≈ 0.1 at both μ* = 0.196 and *λ* = 1.5 and *T*_*c*_/(0.69*Θ*_*D*_) = 0.083 at both μ* = 0.405 and *λ* = 1.5 are obtained. The values of *T*_*c*_/(0.69*Θ*_*D*_) ≈ 0.1 and 0.083 can correspond to (*T*_*c*_/1.14*Θ*_*D*_) ≈ 0.1, the value of the weak coupling-limit of BCS theory. For instance, in the case of *Θ*_*D*_ = 869 K and *T*_*c*_ = 155 K for D_3_S, from *T*_*c*_/(0.69*Θ*_*D*_) ≈ 0.1, an obtained McMillan *T*_*c*_ ≈ 59.96 K is much smaller than *T*_*c*_ = 155 K. Thus, the McMillan *T*_*c*_ does not rise to the room-temperature *T*_*c*_. Moreover, when the strong-coupling correction term of μ* = [(1 − 2*α*)(1 + *λ*)/(1–0.62*λ*)]^0.5^/*ln*(*Θ*_*D*_/1.45*T*_*c*_) is utilized^21^, μ* = 0.223 for both *α* ≈ 0.465^12^, and *λ* = 1.5, and μ* = 0.461 for both *α* ≈ 0.35 and *λ* = 1.5 are calculated. *T*_*c*_/(0.69*Θ*_*D*_) = 0.088 for μ* = 0.223 and 0.014 for μ* = 0.461 are obtained. For example, in the case of *Θ*_*D*_ = 869 K and *T*_*c*_ = 155 K for D_3_S, from *T*_*c*_/(0.69*Θ*_*D*_) ≈ 0.088, a McMillan *T*_*c*_ = 52.77 K, much smaller than *T*_*c*_ = 155 K, is determined. In particular, in the strong coupling, the *T*_*c*_ is smaller than that in the weak coupling. Thus, the McMillan *T*_*c*_ does not approach the room-temperature *T*_*c*_.

### Derivation of the charge density-wave potential, *V*_*CDW*_

For metal, we consider the breathing mode (harmonic oscillation) of an atom, then $E_{Breath} = \frac{1}{2}kx^{2}$, where $k = M\omega^{2}$, *x* is a small deviation from atomic position induced by the oscillation, *M* is a mass of the atom, and $\omega {\text{ is atom}}^{^{\prime}} {\text{s oscillation frequency}}$. Next, for insulator, we consider the breathing mode distortion, $E_{Breath - distortion} = g\delta qx$, where *g* is a proportional parameter, $\delta q = q_{i} - q_{j}$ is a charge disproportionation between nearest neighbor sites. The total Energy, $E_{CDW} = E_{Breath} + E_{Breath - distortion} = \frac{1}{2}kx^{2} + g\delta qx,$ is given. At a condition, $\frac{{dE_{CDW} }}{dx} = 0,{ }x_{0} = - \frac{g\delta q}{k}$ is obtained. When *x* is replaced with $x_{0}$ in $E_{CDW}$, $E_{CDW} = - \frac{{g^{2} \left( {\delta q} \right)^{2} }}{2k} = - \frac{{g^{2} \left( {\delta q} \right)^{2} }}{{2M\omega^{2} }},$ is obtained. On average of *E*_*CDW*_, $< E_{CDW} > = - \frac{{ < g^{2} > \left( {\delta q} \right)^{2} }}{{2M < \omega^{2} > }},$ is given. When *δq* = 0, the electronic structure is one electron per atom of metal. In *δq* = 2*e* case, two electrons are occupied in a site and the nearest neighbor site is empty; this is the bipolaronic system. When *δq* = 1*e*, $< E_{CDW} > = - \frac{{ < g^{2} > }}{{2M < \omega^{2} > }},$ is similar to *λ*/*N*(0) = $\frac{2}{N\left( 0 \right)}\int {\frac{{d\omega \alpha^{2} \left( \omega \right)F\left( \omega \right)}}{\omega } = - \frac{{ < g^{2} > }}{{M < \omega^{2} > }}} ,$ in Eq. (23) (this is also CDW potential) in Ref.^21^ (MacMillan’s paper). When spin is considered, $2 < E_{CDW} > = \frac{\lambda }{N\left( 0 \right)}$ is same. On the basis of this CDW logic, Eq. (23) in Ref.^21^ has an electronic structure in which one electron is occupied in a site and the nearest neighbor site is empty. Then, the number of electrons is half of total electrons in the system, which has a disagreement not satisfied with the metal condition (one electron per atom, that is, half filling) in the normal state (?); this is not bipolaron but just polaron. Finally, we assert that the electron–phonon interaction indicates the CDW interaction.

### Approximate estimation of λ_BCS_

The density of states of sulfur hydride was estimated to be 0.019 states/(spin-eV/Å^3^)^25^, when 2Δ ≈ 30.90 meV in Table 1 is approximately assumed as coupling potential *V*_*e*-*ph*_, λ_BCS_ is given to be 0.587 × 10^–3^. When the density of states is calculated as 0.586 states/(spin-eV/Å^3^)^50^ obtained by assuming the standard BCS relation between energy gap and critical temperature, λ_BCS_ is determined to be 18 × 10^–3^. When, at most, 2Δ ≈ 60 meV is assumed, λ_BCS_ ≈ 36 × 10^–3^ can be evaluated, Thus, we assert λ_BCS_ is very small in an uncorrelated system.

## Supplementary Information

Supplementary Information.
