# Supplementary material for: Room-temperature-superconducting Tc driven by electron correlation
Source: Sci Rep. 2021 May 14;11:10329. doi: 10.1038/s41598-021-88937-7 (PMC8121790; doi:10.1038/s41598-021-88937-7)
Supplement: Supplementary file 1 — Supplementary Information. [file 41598_2021_88937_MOESM1_ESM.docx]

**Supplementary Information for**

**“Room-Temperature-Superconducting *T_c,BR-BCS_* Driven by Electron Correlation”**

Hyun-Tak Kim

Metal-Insulator-Transition Lab., Electronics & Telecommunications Research Institute, Daejeon 34129, South Korea

In this Supplemental Material, we provide detailed derivations of the generalized energy gap of Cooper pair, Eq. (1), and the generalized superconducting *T_c_*, Eq. (2) in BCS theory.

1. **Derivation of the generalized energy gap of Cooper pair.**

First, we find the generalized energy gap of the Cooper pair without a condition of *λ_BCS_=N(0)V_e-ph_* <<1 in BCS theory. The energy gap of Eq. (2.40) in BCS theory [1] is given by using *sinh(x)=(e^x^-e^-x^)/*2 as follows:

$$\varepsilon_{g} \Delta=\frac{\hbar\omega}{\sinh\left[ \frac{1}{\lambda_{BCS}} \right]},$$

$$=\frac{2\hbar\omega}{e^{\left[ \frac{1}{\lambda_{BCS}} \right]}-e^{\left[ -\frac{1}{\lambda_{BCS}} \right]}},$$

$$=\frac{2\hbar\omega}{e^{\left[ \frac{1}{\lambda_{BCS}} \right]}\left[ 1-e^{\left[ -\frac{2}{\lambda_{BCS}} \right]} \right]},$$

$$=\frac{2ћ\omega exp\left[ -\frac{1}{{}_{BCS}} \right]}{1-exp\left[ -\frac{2}{{}_{BCS}} \right]}, (1)$$

where *ћω* is the phonon vibration energy, *λ_BCS_=N(0)V_e-ph_* is the electron-phonon coupling constant when the electron correlation is not considered, *N(0)* is the density of Bloch states of one spin per unit energy at the Fermi surface *E_F_*, and *V_e-ph_* is a constant matrix element of the electron-phonon pair energy. Eq. (1) satisfied with *λ_BCS_* ≠ ∞ has a divergence in the denominator. The Debye temperature *Θ_D_* is *ћω/k_B_.*

1. **Derivation of the generalized superconducting *T_c_***

The *T_c_* equation of Eq. (3.28) in BCS theory [1] is given as

, $z=\frac{\Theta_{D}}{{2T}_{c}}$ is given,

$$\frac{1}{\lambda_{BCS}}=\int_{0}^{z} \frac{tanh(z)}{z}dz,$$

$$\frac{1}{\lambda_{BCS}}=\left[ \tanh\left( z \right)\ln\left( z \right) \right]_{0}^{z}-\int_{0}^{z} \frac{\ln\left( z \right)}{{\cosh\left( z \right)}^{2}}dz,$$

$$=\tanh\left( z \right)\ln\left( z \right)-Integral,$$

$$=\tanh\left( z \right)\left[ \ln\left( z \right)-\coth\left( z \right)\mathrm{Integral} \right],$$

where Integral$=\int_{0}^{z} \frac{\ln\left( z \right)}{{\cosh\left( z \right)}^{2}}dz$.

$$\frac{1}{\lambda_{BCS}\tanh\left( z \right)}=\ln\left( z \right)-\coth\left( z \right)\mathrm{Integral}$$

$$= \ln\left( z \right)+\ln\left\{ \exp\left( -\coth\left( z \right)\mathrm{Integral} \right) \right\}$$

$$=ln\left[ z\left\{ \exp\left( -\coth\left( z \right)\mathrm{Integral} \right) \right\} \right],$$

$$=\ln\left( \mathrm{zC}\left( z \right) \right), (2)$$

where C(z)$=\exp\left( -\coth\left( z \right)\int_{0}^{z} \frac{\ln\left( z \right)}{{\cosh\left( z \right)}^{2}}dz \right)$.

From Eq. (2),

$$\mathrm{zC}\left( z \right)=exp\left( \frac{1}{\lambda_{BCS}\tanh\left( z \right)} \right)\exp\left( \frac{coth(z)}{\lambda_{BCS}} \right),$$

$$,$$

$$\mathrm{zC}\left( z \right) \left( \frac{\Theta_{D}}{{2T}_{c}} \right)C\left( z \right)=exp\left( \frac{\coth\left( z \right)}{\lambda_{BCS}} \right), (3)$$

From Eq. (3),

$$T_{c}=C\left( z \right)\Theta_{D}exp\left[ -\frac{\coth\left( z \right)}{\lambda_{BCS}} \right], (4)$$

is derived.

In order for *T_c_* in Eq. (4) to be maximum, C(z) should be maximum, then z must be satisfied with z = ∞ in the integral *C(z)*.

Accordingly,

$$C\left( z \right)\equiv\frac{1}{2}exp\left[ -\int_{0}^{\infty} (ln(z)/{cosh}^{2}z)dz \right]=\left( \frac{2e}{} \right)1.13,$$

is determined, where γ≈0.577 is the Euler constant.

Finally, the generalized *T_c_*,

$$T_{c} 1.13\Theta_{D}exp\left[ -\frac{\coth\left( z \right)}{\lambda_{BCS}} \right], (5)$$

is determined.

Although Eq. (5) was first performed [2], but, because of the compressed derivation process, a confirmation of Eq. (5) was difficult. Therefore, I easily re-derive it for its confirmation and readers.

1. **Physical meaning of the BR-BCS *T_c_* of Eq. (6)**

Figure below explains the physical meaning of Eq. (6). Let’s consider the percolation in an inhomogeneous sample. As pressure increases, metallization increases by continuously generating the insulator-metal transition such as domains 1 -> 2 -> 3 -> 4 -> 5. The respective domains in Fig. (a) have impurities of n_c_, an impurity critical carrier density, inducing the insulator-metal transition. The insulator-metal transition occurs by excitation of n_c_ by applying pressure. And let’s consider only domain 1. When A GPa is applied to the sample, the insulator domain 1 (Fig. (a)) becomes metal (Fig. (b)). The metal (Fig. (b)) becomes a superconductor with the true *T_c_* of Eq. (7). However, let’s consider whole sample. The metal region of domain 1 in the whole sample is ρ1, then the true *T_c_* of Eq. (7) for domain 1 should be averaged in the whole measurement region of the sample, which follows Eq. (6).


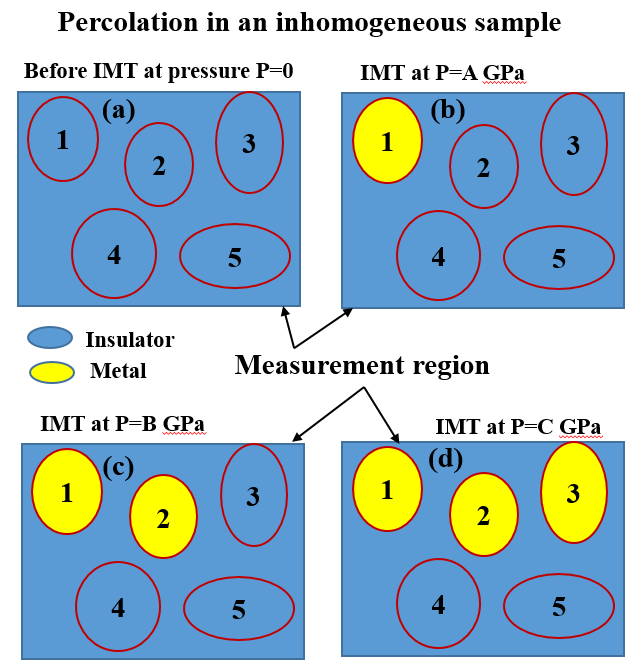


Figure 1: a percolation model explaining the physical meaning of the BR-BCS Tc.

References

1. Bardeen, J., Cooper, L. N. & Schrieffer, J. R. Theory of Superconductivity, [Phys. Rev. 108, 1175 (1957)](https://journals.aps.org/pr/abstract/10.1103/PhysRev.108.1175).

2. Aguilera-Navarro, V. C. & de Llano, M. High-T_c_ BCS gap-to-T_c_ ratio[, International Nuclear Information System (INIS) Vol. 22, No 19, 10 (1991)](https://inis.iaea.org/search/search.aspx?orig_q=RN:22067860).
